# Basal paravian functional anatomy illuminated by high-detail body outline

**DOI:** 10.1038/ncomms14576

**Published:** 2017-03-01

**Authors:** Xiaoli Wang, Michael Pittman, Xiaoting Zheng, Thomas G. Kaye, Amanda R. Falk, Scott A. Hartman, Xing Xu

**Affiliations:** 1 Institute of Geology and Paleontology, Linyi University, Linyi City, Shandong 276005, China; 2 Vertebrate Palaeontology Laboratory, Department of Earth Sciences, University of Hong Kong, Pokfulam, Hong Kong, China; 3 Shandong Tianyu Museum of Nature, Pingyi, Shandong 273300, China; 4 Foundation for Scientific Advancement, 7023 Alhambra Drive, Sierra Vista, Arizona 85650, USA; 5 Department of Biology, Centre College, 600 West Walnut Street, Danville, Kentucky 40422, USA; 6 Department of Geoscience, University of Wisconsin-Madison, Lewis G. Weeks Hall for Geological Sciences, 1215 West Dayton Street, Madison, Wisconsin 53706-1692, USA; 7 Key Laboratory of Vertebrate Evolution and Human Origins of Chinese Academy of Sciences, Institute of Vertebrate Paleontology and Paleoanthropology, Chinese Academy of Sciences, Beijing 100044, China

## Abstract

Body shape is a fundamental expression of organismal biology, but its quantitative reconstruction in fossil vertebrates is rare. Due to the absence of fossilized soft tissue evidence, the functional consequences of basal paravian body shape and its implications for the origins of avians and flight are not yet fully understood. Here we reconstruct the quantitative body outline of a fossil paravian *Anchiornis* based on high-definition images of soft tissues revealed by laser-stimulated fluorescence. This body outline confirms patagia-bearing arms, drumstick-shaped legs and a slender tail, features that were probably widespread among paravians. Finely preserved details also reveal similarities in propatagial and footpad form between basal paravians and modern birds, extending their record to the Late Jurassic. The body outline and soft tissue details suggest significant functional decoupling between the legs and tail in at least some basal paravians. The number of seemingly modern propatagial traits hint that feathering was a significant factor in how basal paravians utilized arm, leg and tail function for aerodynamic benefit.

## Abstract (web summary)

Soft tissues are rarely preserved in the fossil record; therefore, body shape of extinct vertebrates is usually inferred indirectly. Here, the authors use laser-stimulated fluorescence of fossils to detect and reconstruct the body outline of the paravian dinosaur *Anchiornis* from the Late Jurassic.

Laser-stimulated fluorescence (LSF) imaging can broaden the scope of data available from fossils by revealing morphological details that are otherwise invisible under white or ultraviolet light conditions^1^. In this study, LSF imaging is performed on the four-winged dinosaur *Anchiornis*^2^,^3^, one of a few key basal paravian theropods—including *Microraptor* and *Archaeopteryx*—whose osteology, feathering and aerodynamics have made profound contributions to the understanding of avian origins and early flight evolution^3^,^4^,^5^,^6^,^7^,^8^. *Anchiornis* is especially suited for this study because its contribution to understanding avian and flight origins has not been fully realized. As the earliest four-winged paravian with long tibial and metatarsal feathers, *Anchiornis* shares a key feature with gliding-capable *Microraptor*^3^,^5^,^9^. *Anchiornis* is also represented by 229+ specimens^10^, which greatly improves the chance of discovering rare soft tissue preservation with minimal taphonomic damage.

The LSF data collected was used to produce and describe the quantitative outlines of portions of the fossil paravian body. In providing much needed fossilized soft tissue evidence, the scope of indirect skeletal and feather evidence in determining basal paravian body shape will be better understood. Additionally, the utility of body shape inferences based on an extant phylogenetic bracket (EPB) approach can be gauged. The reconstructed leg and tail outlines of *Anchiornis* suggest significant functional decoupling between these body parts, as was likely the case in many other basal paravians. The traits of the reconstructed patagia-bearing arms hint that feathering was a significant factor in how basal paravians utilized their appendages for aerodynamic benefit.

## Results

### Body outline

**Figure 1:**
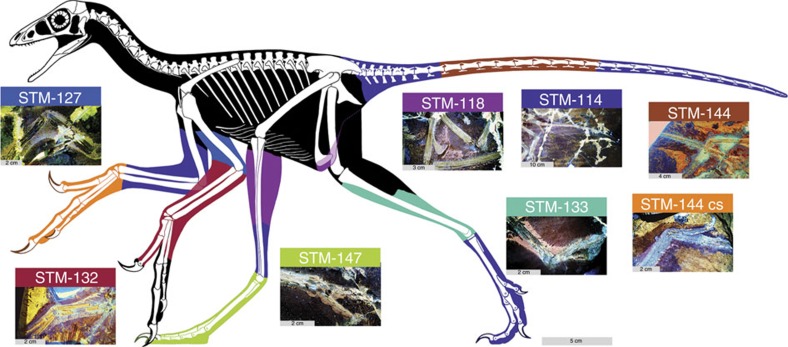
Reconstructed body outline of basal paravian *Anchiornis* using LSF images. Coloured areas represent different specimens and black ones are approximated from equivocal data. Skeleton predominantly reconstructed from STM-118 and its scale is 5 cm. The inset specimen images have different scales: in STM-127, 132, 147 and 144CS it is 2 cm, in STM-118 it is 3 cm, in STM-144 it is 4 cm and in STM-114 it is 10 cm. See Methods.

LSF imaging recovered high fidelity outlines of the arms, legs and tail for *Anchiornis*, but head, neck and thorax outlines could not be reliably imaged (Fig. 1). The body parts imaged under fluorescence were highly representative of the original tissues, but no chemical analysis was done to determine if they were organic or mineral in nature. For descriptive purposes in this paper, standard anatomical references are used until such time that a thorough analytical assessment of the preservation allows for more accurate vernacular.

### Forelimb outline

**Figure 2:**
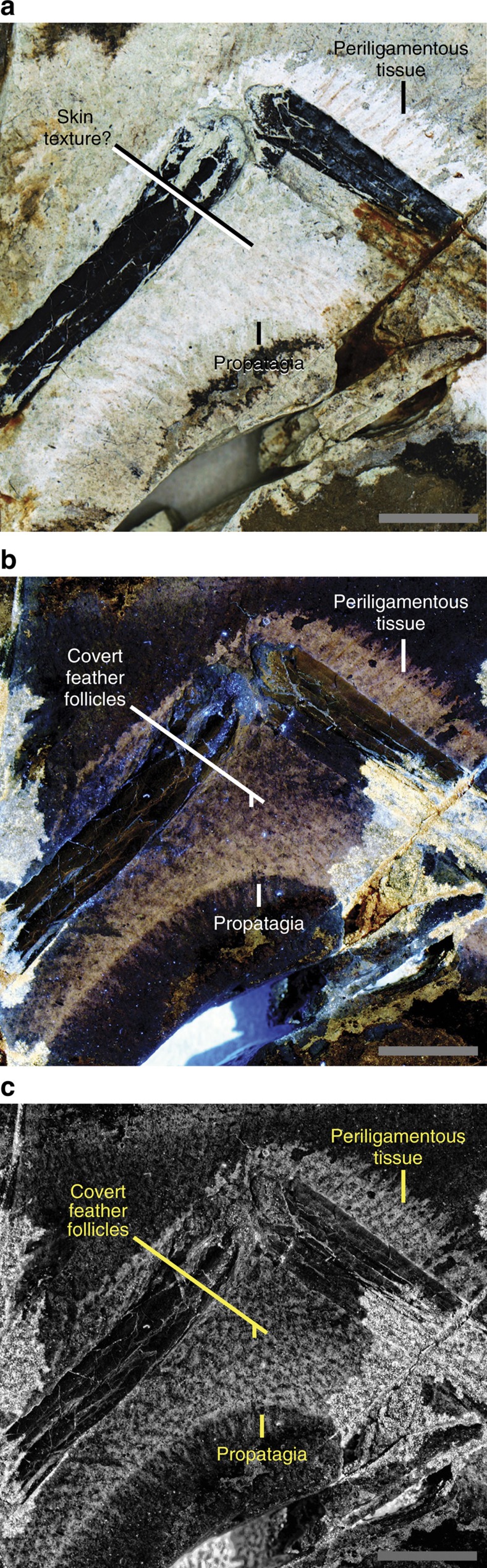
Shallow propatagium of *Anchiorni*s STM-0-127 at 95° of elbow extension. Almost regularly spotted skin texture are covert feather follicles. Scales are 1 cm. (**a**) White light image, (**b**) LSF image and (**c**) LSF image with rank filter applied.

The slender arm outline is widest across the propatagia (Figs 1 and 2). The latter are preserved at 69°–94° of elbow extension and are less widely extended than the smaller postpatagia (Supplementary Fig. 1; Supplementary Table 1). There is no obvious alular patagium, unlike enantiornithine MCCMLH31444 (ref. 11). The propatagial surface is covered in almost evenly spaced—but not scattered^11^—spots that occasionally contain brown filamentous structures. The third manual digit is covered in small rounded reticulate scales (Supplementary Fig. 2). The outline interpreted as the margin of the soft tissue is approximately twice as thick as the phalanges of the third digit and has a smooth ventral surface without thick fleshy pads, as in the fingers of oviraptorosaur *Caudipteryx* (Fig. 1, plate II of ref. 12).

### Hindlimb outline

**Figure 3:**
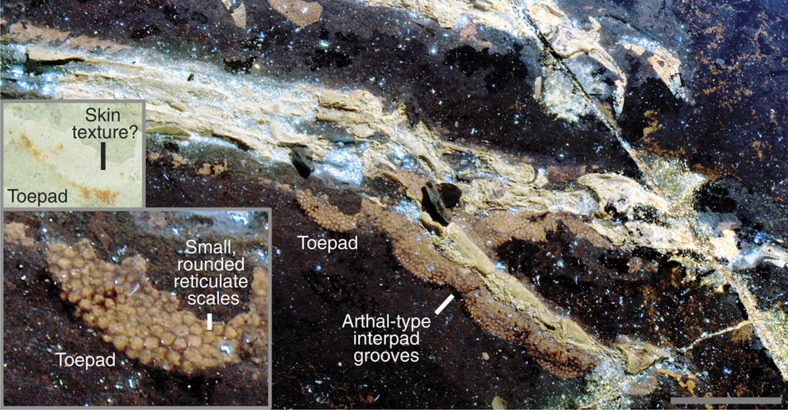
Plantar footpads of *Anchiornis* STM-0-147. These preserve reticulate scales and arthal-type interpad grooves. Scale is 1 cm. Inset images of a footpad under white and laser light.

The leg outlines are known up to the proximal end of the tibia and fibula and are widest at the proximal two-thirds of the latter (Fig. 1), the same shape found in birds^13^,^14^. The feet have bird-like plantar footpads with interpad grooves exhibiting the typical arthal condition of theropods^15^,^16^ (Figs 1 and 3). The footpads are covered in pebble-shaped reticulate scales (Fig. 3), but the dorsal foot scales are minimally discernible. There are pebble-shaped tibia scales and equivocal ankle scales (Supplementary Fig. 3).

### Tail and pelvic outline

**Figure 4:**
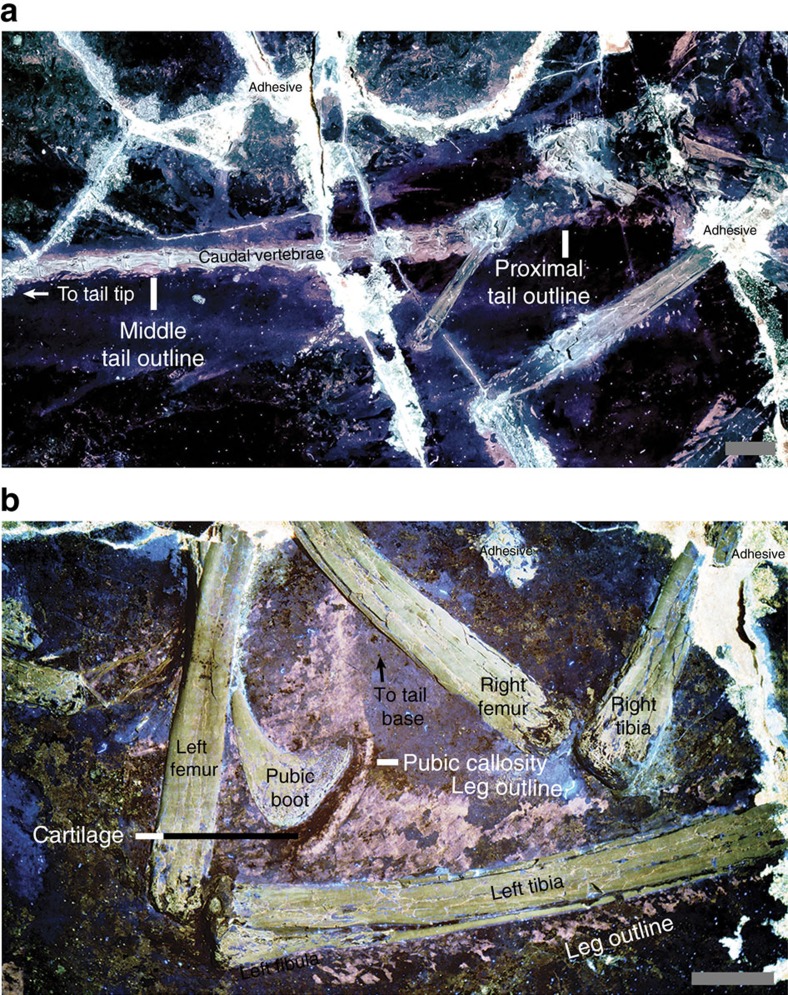
Tail outline and outline of leg and pubic boot. (**a**) Tail outline of STM-0-114. (**b**) Left leg and pubic boot outline of STM-0-118. The latter forms a potential cartilage-supported (black band) callosity. Scales are 1 cm.

The slender tail outline hugs the caudal vertebrae, but at its base it forms a gently concave margin from the third caudal to the region around the posterior ramus of the ischium (Figs 1 and 4a; the ischium is not preserved in STM-0-114, but its position is inferred from STM-0-118 and other specimens). The body outline follows the shape of the pubic boot and then ascends at a steep angle towards the distal end of the ischium.

The bird-like body outline reconstruction in Fig. 1 confirms existing skeleton and feather-based inferences and supports EPB-based studies of leg and tail shape^17^,^18^. The drumstick-shaped legs of *Anchiornis* (Fig. 1), and probably of most theropods, are concordant with the relatively slender foot and distal portion of the tibia and fibula as well as the broader proximal portion of the latter bones and the robust femur. The slender tail (Figs 1 and 4a) also fits the shallow and narrow caudal centra and their shallow chevron depths. The observed leg and tail outline are also in agreement with reconstructed theropod musculature using fossil bones studied through an EPB-based approach, validating this method. However, future studies will benefit from the new data in helping to better constrain leg muscles such as the M. fibularis longus and M. gastrocnemius lateralis and tail muscles such as the M. transversospinalis and M. ilio-ischio caudalis^13^,^17^,^18^,^19^,^20^,^21^ (Figs 1 and 4). Scales from the tibia to the feet suggest that *Anchiornis* had podotheca like modern birds^15^ and other tetanurans^13^,^16^ (Supplementary Fig. 3). The toe pads in particular are of modern avian aspect (Figs 1 and 3). Between the tail and legs, there is a potential cartilage-supported pubic callosity that may have been suited for partially supporting the animal as it rested on the ground (Fig. 4b). However, it could simply indicate that the individual (STM-0-118) had yet to reach maturity, although there are no clear indications of ontogenetic stage elsewhere in the specimen.

## Discussion

The *Anchiornis* propatagia observed in this study are the first direct examples among four-winged dinosaurs (see Supplementary Note 1 for a purported example in *Archaeopteryx*^22^) and were associated with symmetrical feathering in life^3^. They show shape discrepancies with the halos preserved in the matrix around the bones, suggesting that the latter should not be taken at face value as fossilized soft tissues without complementary evidence such as preserved feathering (*Microraptor*: Supplementary Figs 2 and 4; Fig. 2 of ref. 23). Modern avian propatagia form the leading edge of the aerofoil and help give it a cambered profile^24^. Propatagia make major contributions to lift generation proximal to the wrist without which birds cannot fly^25^. This function may have been possible in *Anchiornis* given that we already know that the movement of some four-winged dinosaurs through the air was limited by wing area^6^, a property that propatagia help to increase. However, some living flightless carinate birds have similar propatagial muscle complexes to their volant relatives^26^. Propatagia also have a deep non-flight-related non-avian theropod origin for example, those in *Caudipteryx* (Fig. 4 of ref. 23; see Supplementary Note 1). Specimens STM-0-114, 127 and 132 have the best preserved propatagia and their shallow depth (1.2–1.5 cm) even at obtuse elbow angles indicates that the arm was not nearly fully extended when these individuals died. This implies that the propatagium was kept taut either by a form of ligamentum propatagiale^27^,^28^ or by other portions of the propatagial muscle complex. The propatagia also suggest that *Anchiornis* could produce a relatively straight arm, a posture broadly found in many living gliding birds (for example, comorants, albatrosses and pelicans). This indicates a previously unknown aspect of arm morphology differentiation at the earliest stages of paravian evolution (at least by the Oxfordian stage of the Late Jurassic^3^) that may even have been widespread. The aforementioned differentiation in arm morphology among basal paravians implies functional diversity that is in keeping with the diversity of arm feathers seen in four-winged paravians: symmetrically vaned in *Anchiornis*^3^ and asymmetrically vaned in *Microraptor* and *Archaeopteryx*^5^,^9^. It also complements differences in the muscular control of basal paravian arms as implied by the presumably weaker muscle attachments to the non-ossified sterna of *Anchiornis* and *Archaeopteryx*, and presumably stronger muscle attachments to the ossified sterna of *Microraptor*^10^. The uncrossed and skin-bound second and third manual digits of *Anchiornis* (Fig. 1, Supplementary Fig. 5) formed a functionally didactyl hand, as in Enantiornithes^11^. The latter trait presumably helped to stiffen the postpatagium, but it is unclear whether it was present in other four-winged dinosaurs too (for example, *Microraptor*: Supplementary Fig. 4). A stiffer feathered postpatagium in *Anchiornis* may have compensated for its aerodynamically inferior arm feathers to some degree, providing another example that basal paravians may have evolved multiple solutions to similar locomotor needs and challenges. Improved understanding of the interactions between functional parameters is, therefore, a crucial step towards a more holistic understanding of basal paravian function. However, it is clear that there was a range of functional capabilities among basal paravian arms, including those used for aerodynamic purposes.

The well-preserved propatagial surface of *Anchiornis* (Fig. 2) shows the earliest known details of paravian covert feather attachment and arrangement^11^,^29^. In *Anchiornis*, regularly-spaced spots on the surface of the propatagium are interpreted as covert feather follicles (Fig. 2). These are not arranged in tracts—unlike modern birds—indicating little feather differentiation as seen in its plumage^3^. These are not closely packed near the leading edge of the wing which was covered in covert feathers that were comparatively longer than those of most living birds (Supplementary Fig. 5; ref. 13; Fig. 1 of ref. 24). However, the diagonal orientation of these coverts (Fig. 1, Supplementary Fig. 5) is shared with living birds^24^, indicating some degree of leading edge camber in the wings of *Anchiornis*. This camber is supported by the denser colouration of the propatagial leading edge, a two-dimensional representation of the original three-dimensional morphology^30^ (Fig. 2). Brown-coloured filamentous structures associated with some of the feather follicles appear to be fossilized *in situ* periligamentous tissue (Fig. 2). These filaments suggest that these animals shared robust feather attachments (Fig. 13 in ref. 24) with some Early Cretaceous birds^11^,^29^ (and probably many feathered non-avian dinosaurs as well). It is probable that other aspects of the modern avian dermal system^16^ were already present in basal paravians, but this requires further investigation. Current taxonomy refers *Anchiornis* to basal Paraves^2^,^3^,^8^ and it has also been referred to Averaptora^28^. Future soft tissue studies such as this one promise to further refine our understanding of paravian patagial evolution.

The symmetry of the arm feathers and their regular, non-tract-based arrangement suggest that the arms were probably not used in a comparably way to modern birds, despite striking similarities in propatagium shape and camber. This suggests that feathering—particularly its symmetry, size differentiation and spatial arrangement—was highly significant towards how some basal paravians utilized their arm (and likely leg and tail) function for aerodynamic benefit.

Functional independence between the hind limb and tail of living birds enables them to finely control their respective flight surfaces and is the result of a continuous transition from hip to knee-driven locomotion along the theropod lineage to birds^17^,^18^. The new hind limb and tail outline results support the absence of a large *M. caudofemoralis* in *Anchiornis*, as predicted by bone morphology^17^,^18^. This indicates that *Anchiornis* had finer and more independent control of its hind limb and tail (and the feathers attached to them) compared with more basal theropods^17^,^18^, although the detailed implications of these new hind limb and tail constraints—particularly in comparing the hind limb and tail function of basal paravians with that of modern birds—requires future investigation using quantitative biomechanical modelling.

The reconstructed body outline of *Anchiornis* is expected to be similar to asymmetrically feathered basal paravians like *Microraptor* and *Archaeopteryx* based on their skeletal similarities. This body outline study supports the modelled aerodynamic performance of basal paravians^4^,^6^ and promises to reconstruct even greater prowess in the future. This work, therefore, builds a strong foundation for determining the aerodynamic capabilities of *Anchiornis* be it ground-based or airborne^3^,^7^.

## Methods

### Materials and taxonomy

The body outline in Fig. 1 and the other figures in the manuscript show the details of nine basal paravian specimens (STM-0-7, 114, 118, 125, 127, 132, 133, 144 and 147) that can all be referred to *Anchiornis*. These specimens are deposited in the Shandong Tianyu Museum of Nature in Pingyi, China. *Anchiornis* has been referred to a basal bird^2^, a basal troodontid^3^,a basal deinonychosaur^8^ or an averaptoran^28^ so in the absence of taxonomic consensus it is referred more simply to a basal paravian in this study. Two diagnostic features of *Anchiornis huxleyi* were included in the original description^2^ (IVPP V14378, housed at the Institute of Vertebrate Paleontology and Paleoanthropology in Beijing, China): extreme shortness of the ischium and a sculpturing pattern of numerous small pits on the ventral surface of the coracoid. The ventral position of the latter makes it a difficult feature to observe so it is no surprise that it has yet to be confirmed in other *Anchiornis* specimens and in the nine aforementioned specimens. An extremely short ischium is observed in STM-0-7, 118, 127 and 132, but this character is not exposed in the other five specimens figured in the manuscript. Unlike *Xiaotingia*, metacarpal III is thinner than metacarpal II in all of the body outline specimens. Unlike *Eosinopteryx*, STM-0-114, 125 and 132 have more than 23 caudal vertebrae, but this is uncertain in the six other figured specimens. Unlike *Aurornis*, no evidence of an elongate metatarsal I is observed in the body outline specimens, although in STM-0-133 the metatarsals are not well exposed. The overall similarities in the exposed portions of all of the body outline specimens indicate that these individuals can be referred to *Anchiornis* (for example, a short deltopectoral crest, a straight ulna, and so on.); these characters were also used to refer LPM-B00169 to this genus^3^ (housed at the Liaoning Paleontological Museum in Shenyang, China).

### LSF imaging protocol and theory

LSF images were collected using the protocol of Kaye *et al*.^1^. *Anchiornis* specimens were imaged with 405 and 532 nm, 500 mw lasers. An appropriate long pass blocking filter was used in front of the camera lens to prevent image saturation by the laser. The laser was projected into a vertical line by a Laserline Optics Canada lens, which was mechanically swept repeatedly over the specimen during the photo's time exposure in a dark room. The images were post processed in Photoshop for sharpness, colour balance and saturation.

Fluorescence emanates from luminescent centres in the mineral lattice^31^. The mineral lattice is not pure but incorporates organic and inorganic molecules into the lattice at the time of formation^32^. The luminescent centre contains the contaminating molecule in close association with the electron clouds of the mineral^31^. Photons entering the lattice statistically transfer their energy to the electron clouds and cause changes in electron excitation levels. The excited vibrational energy states of the electrons then decay through multiple random paths some of which emit photons while others do not. The excitation and decay process is defined by ligand field theory which applies group theory and quantum mechanics to electrostatic theory^31^. A full description of ligand field theory is beyond the scope of this paper. Pertinent to fossil analysis, the contaminants of the mineral structure are typically on a parts-per-million basis, which makes fluorescence a sensitive analytical tool^33^. A colour change represents a different electron decay process from a different atomic arrangement^31^. Due to the complexity of the decay path, specific colours cannot be attributed to a particular molecular arrangement just from an image^31^. However, differences in colours do represent changes in the luminescent centre's makeup. Additional complex laboratory analysis can determine the nature of the luminescent centre which is the target of further study.

### Skeletal reconstruction

The skeletal reconstruction was illustrated in Adobe Photoshop CC 2015. Individual bones were scaled from high-resolution photographs exhibiting minimal parallax using the Photoshop Ruler Tool. The virtual scaling was set according to scale bars photographed in the same plane as the specimens, using Photoshop's Custom Scaling tool (Image -> Analysis -> Set Measurement Scale). Bones were illustrated so that individual measurements end at the edge of the white portion of the bone, as opposed to the middle or outside of the black bounding line. The skeletal diagram is based primarily on STM-0-118. Missing caudal elements were cross-scaled from STM-0-114. Major elements were illustrated on separate layers to facilitate rotation and transformation into plausible life positions. Presacral vertebrae provided the lengths and representative heights of vertebral elements, but were not preserved with the lateral aspect visible in sufficient quantities to determine the exact curvature of articulation in the neck and back. Articulated and well-exposed presacral series of basal paravians (for example, *Jinfengopteryx*) were used as supplementary guides for reconstructing vertebral curvature in *Anchiornis*. Forelimb elements were articulated following the left forelimb of STM-0-144, which is preserved with joint angles consistent with contemporary biomechanics work. Hind limb elements of STM-0-118 and STM-0–114 were preserved in articulation, and in good accordance with published interpretations of theropod limb kinematics^17^,^18^, and were the basis for the pose in Fig. 1.

### Soft tissue reconstruction

Examples of soft-tissue preservation were inspected for a lack of continuity and evidence of taphonomic distortion. To reduce discrepancies between specimens of differing size, tissue depth was measured as a percentage of bone width or length. Soft-tissue remains represented by multiple specimens exhibiting similar depth and no signs of distortion were taken at face value and reproduced directly in Fig. 1, including most distal limb elements. The propatagium depth at the elbow varied in specimens based on the angle of the elbow, a condition also seen in extant birds^24^,^25^, so the depth reconstructed in Fig. 1 was matched to specimens of similar degrees of elbow flexion. Soft-tissue elements that showed some degree of distortion were used as a qualitative guide for reconstructing the remaining soft-tissue silhouette (represented in black in Fig. 1), and other portions were based on dissections of birds and published theropod myology^13^,^20^. We assigned anatomical terms of soft tissues based on the morphology we observed, in accordance with traditional morphological interpretations of vertebrate fossils (for example, bones, scales, feathers, propatagium).

### Data availability

The data that support the findings of this study are available from the corresponding author upon reasonable request. The data reported in this paper are detailed in the main text and in the Supplementary Information.

## Additional information

**How to cite this article:** Wang, X. *et al*. Basal paravian functional anatomy illuminated by high-detail body outline. *Nat. Commun.* **8,** 14576 doi: 10.1038/ncomms14576 (2017).

**Publisher's note**: Springer Nature remains neutral with regard to jurisdictional claims in published maps and institutional affiliations.

## Supplementary Material

Supplementary Information Supplementary Figures, Supplementary Tables, Supplementary Notes and Supplementary References
