# Supplementary material for: Basal paravian functional anatomy illuminated by high-detail body outline
Source: Nat Commun. 2017 Mar 1;8:14576. doi: 10.1038/ncomms14576 (PMC5339877; doi:10.1038/ncomms14576)
Supplement: Supplementary Information — Supplementary Figures, Supplementary Tables, Supplementary Notes and Supplementary References [file ncomms14576-s1.pdf]

Supplementary Note 1

Existing pennaraptoran propatagia. In addition to *Anchiornis*, preserved propatagial skin and/or their convert feathers are known from the following basal paravians:

Avialae

- Basal avialan *Archaeopteryx* - purported mould of propatagium on the Berlin specimen (Fig. 2 of [1,2]). During direct study of the specimen, two of the authors (MP and TGK) were unable to dismiss this feature as an artefact of fossil preparation.
- Confuciusornithid *Confuciusornis* - preserved propatagial skin and feathering (Fig. 3 of [2])
- Enantiornithine *Noguerornis* - preserved covert feathers (Figs. 10.2, 10.3 of [3])
- Enantiornithine MCCMLH31444 - preserved propatagial (and postpatagial and alular) skin and feathering (Fig. 1 of [4])
- Enantiornithine hatchlings DIP-V-15100, -15101 - preserved propatagial skin and feathering (Figs. 1-3 of [5])
- Scansoriopteryid *Scansoriopteryx* - purported propatagial skin[2]

Dromaeosauridae

- Microraptorine *Microraptor* - purported propatagial skin (Fig 2. of [2]; Supplementary Fig. 4)

Oviraptorosauria

- Oviraptorosaur *Caudipteryx* (preserved propatagial skin and feathering: Fig. 4 of [2])

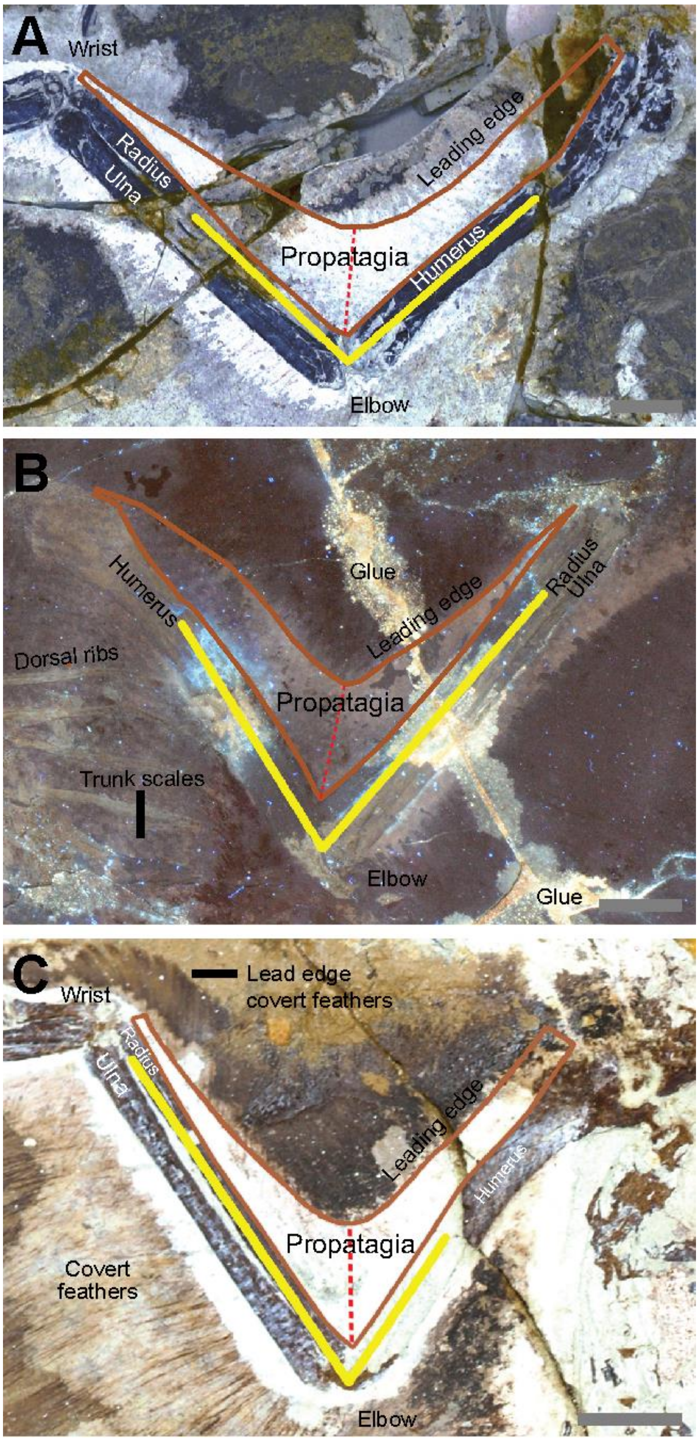

Supplementary Figure 1.

Measurement landmarks in *Anchiornis* propatagia preserved at different degrees of elbow extension. (**A**) STM-0-127 under white light, (**B**) STM-0-114 under laser light and (**C**) STM-0-144 counter slab under white light. The yellow lines demarcate the landmarks used to measure elbow extension - the medial surfaces of the radius and humerus. The red stippled lines shows where propatagium depth was measured - between the leading edge of the propatagium and the dorsal surface of the elbow joint. The brown lines enclose the propatagium area measured - the entire area preserved between the wrist joint and the middle-height of the deltopectoral crest. Scales are 1cm. See Supplementary Table 1 for measurements.

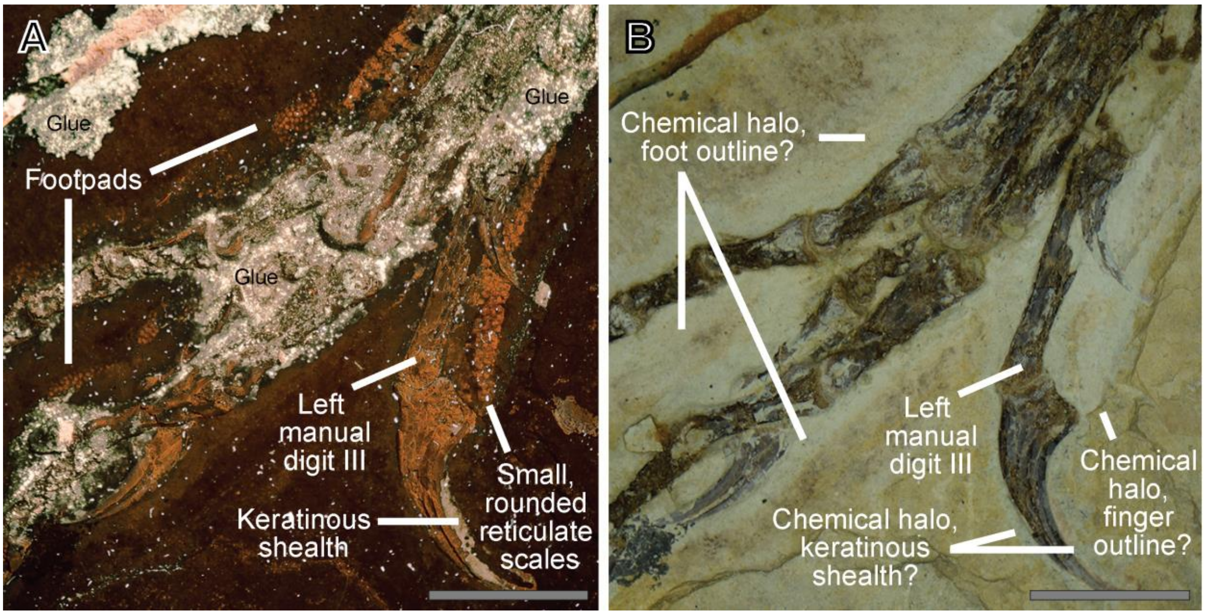

Supplementary Figure 2.

Scaley skin on the fingers of *Anchiornis*. Left manual digit III of STM-0-7 preserves small rounded reticulate scales along its ventral surface. This is a rarity among basal paravians and is the earliest record of it. A foot is preserved beneath the left hand (to the far left). Scale is 1cm. (**A**) LSF image and (**B**) white light image. Note the shape discrepancy between the finger outline in the LSF image compared to the chemical halo visible under white light. This suggests that chemical halos should not be taken at face value when inferring soft tissue anatomy in fossils.

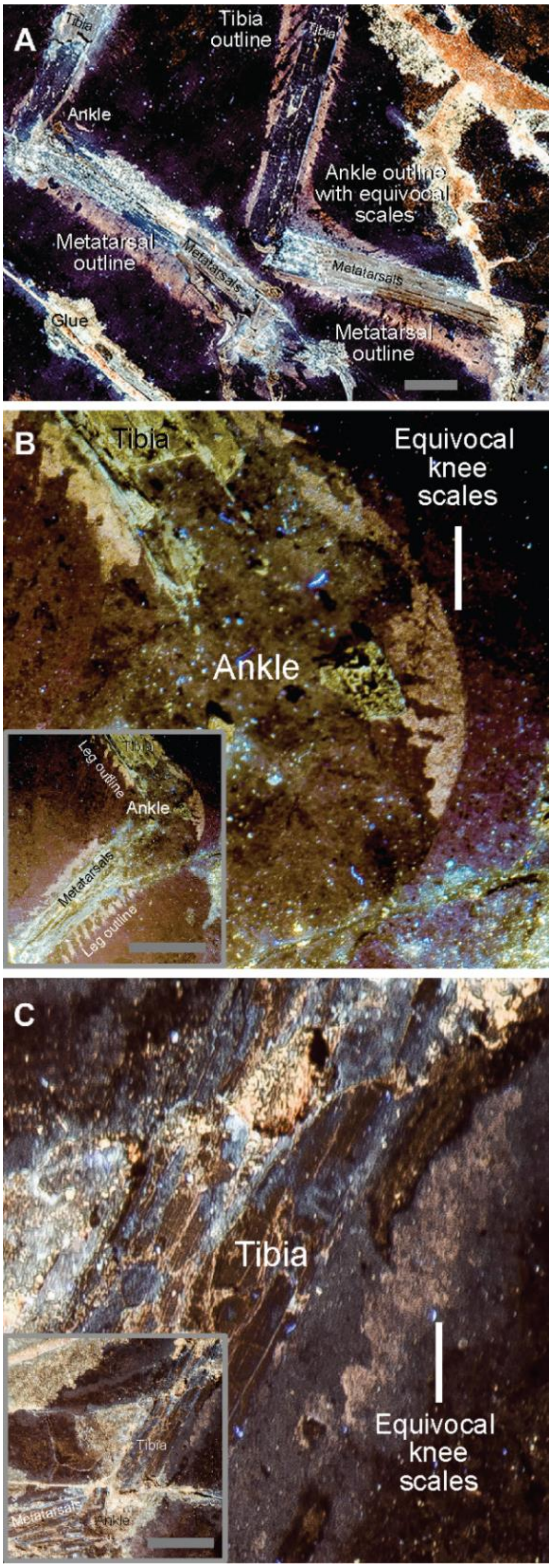

Supplementary Figure 3.

Equivocal ankle scales and preserved tibia scales in *Anchiornis*. (**A**) Equivocal scales on the front of the ankle of STM-0-114, (**B**) equivocal scales on the back of the ankle of STM-0-133 (inset shows zoomed out view), and (**C**) small, rounded scales are preserved behind the tibia of STM-0-125 (inset shows zoomed out view). All scales are 1 cm and all images were taken under laser light.

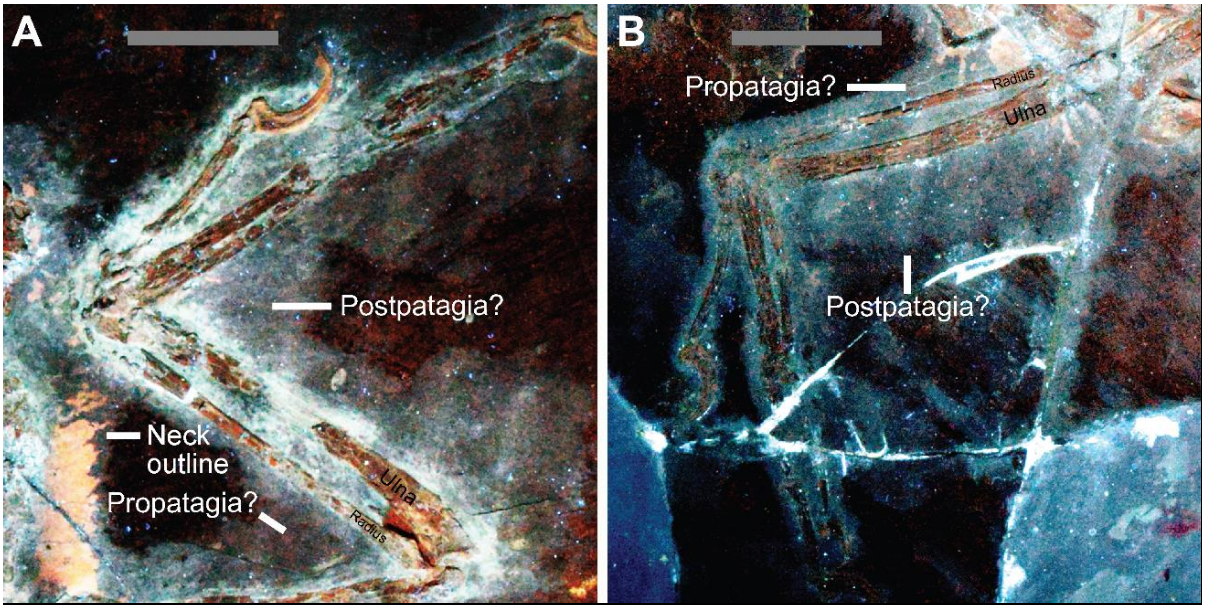

Supplementary Figure 4.

LSF images of the iconic wings of *Microraptor* IVPP V13352. The wings lack well-preserved propatagia, although they were probably present, as in other pennaraptorans (see supplementary information: existing pennaraptoran propatagia). (**A**) Right wing. (**B**) Left wing. Scales are 1cm.

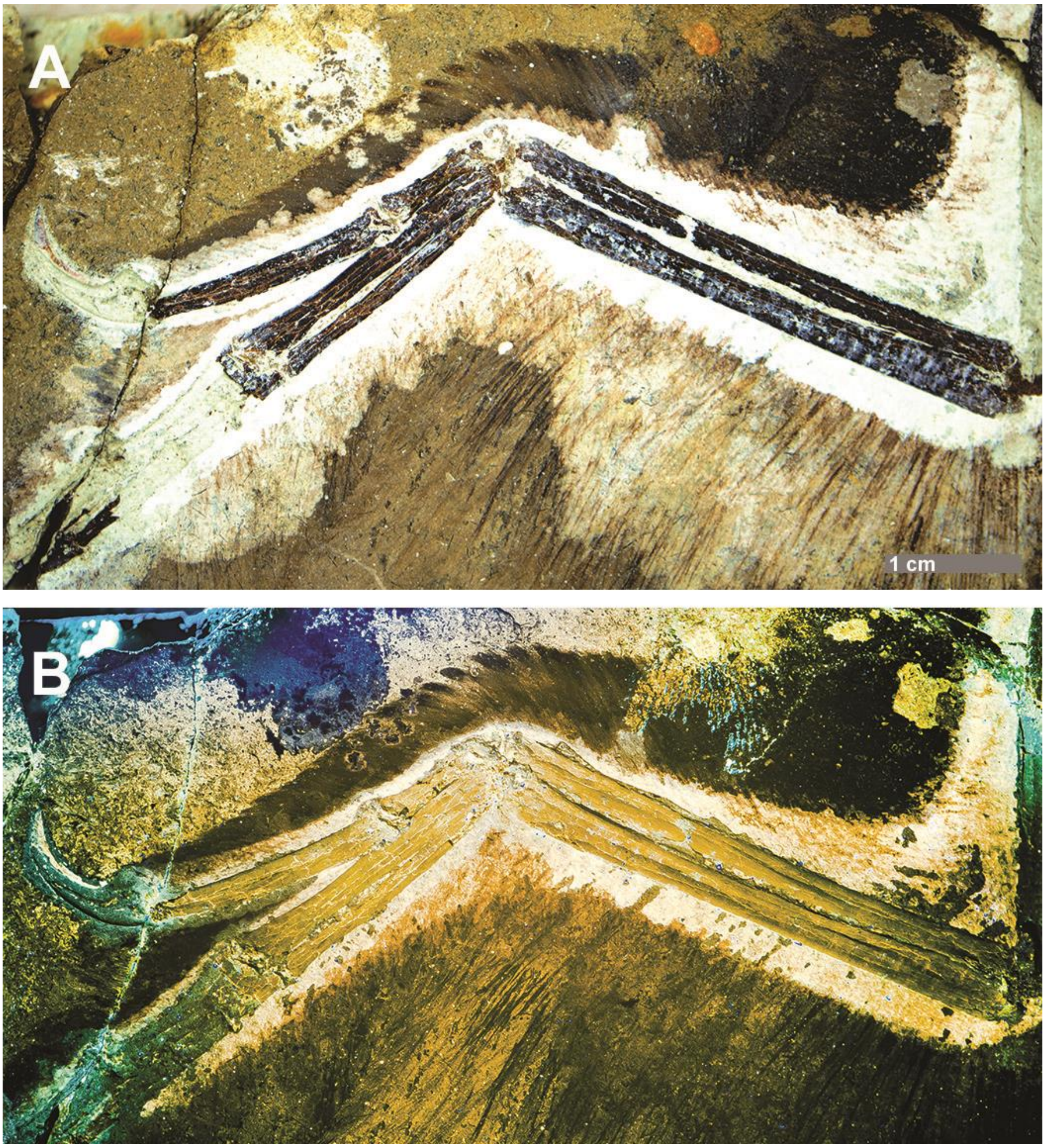

Supplementary Figure 5.

Well-preserved wing of *Anchiornis*. Specimen STM-0-144 counter slab shows uncrossed and skin-bound second and third manual digits, as in enantiornithines[4,6]. This probably helped to stiffen the wing and improve its aerodynamic performance. It also shows relatively long diagonally orientated leading edge coverts that suggests a degree of leading edge wing camber, even they are displaced from their life position. (**A**) White light image and (**B**) LSF image. Scales are 1cm.

Supplementary Table 1. Anatomical features pertinent to the identification of *Anchiornis*. Key: a = *Anchiornis*-like feature; n = non-*Anchiornis*-like feature; ? = cannot determine; - = not observable.

	Specimen number (STM-0-X)								
**Anatomical feature**	**7**	**114**	**118**	**125**	**127**	**132**	**133**	**144**	**147**
Straight premaxilla	a	-	a	-	-	a	-	a	a
Mandible shape	a	-	a	-	-	a	-	a	a
External mandibular fenestra absent	-	-	a	-	-	a	-	-	a
> 23 caudal vertebrae, unlike *Eosinopteryx*	-	a	-	a	-	a	-	-	-
Ischium greatly reduced in length	a	-	a	-	a	a	-	?	-
Short deltopectoral crest of humerus	a	a	a	a	-	-	a	a	-
Ulna and radius straight	a	a	a	a	-	a	a	a	a
Proximal fibular expanded significantly	a	?	a	a	a	a	-	a	-
Unexpanded distal tibia	a	-	a	a	a	a	a	a	a
10th-12th caudals reach maximum length, unlike *Eosinopteryx*	a	a	a	a	?	a	-	a	?
Pedal digit III-1 less than 1.5 times the length of II-1, unlike *Pedopenna*	-	a	a	-	a	a	-	a	a
No evidence of long metatarsal I, unlike *Aurornis*	a	a	a	a	a	a	-	a	a
Metacarpal III thinner than metacarpal II, unlike *Xiaotingia*	a	a	a	a	a	a	a	a	a

Supplementary Table 2. Propatagium measurements from three exquisite specimens (STM-0-9, 0-114 and 0-127). Elbow extension was measured as the angle between the medial surface of the radius and humerus. Propatagium depth was measured as the distance between the leading edge of the propatagium and the dorsal surface of the elbow joint. Propatagium area was measured between the wrist joint and the middle-height of the deltopectoral crest. See Fig. S1 for measurement landmarks.

STM specimen	Elbow extension/°	Propatagium depth/cm	Propatagium area/cm^2
0-9	68.5	1.19	2.88
0-114	73.0	1.40	4.18
0-127	95.3	1.52	6.91

Supplementary References

1 Martin, L. D. & Lim, J. D. Soft body impression of the hand of *Archaeopteryx*. *Current Science* **89**, 1089-1090 (2005).

2 Feduccia, A. & Czerkas, S. A. Testing the neoflightless hypothesis: propatagium reveals flying ancestry of oviraptorosaurs. *Journal of Ornithology* **156**, 1067-1074 (2015).

3 Chiappe, L. M. & Lacasa-Ruiz, J. in *Mesozoic birds: above the heads of dinosaurs* (eds L.M. Chiappe & L. Witmer) 230-239 (University of California Press, 2002).

4 Navalón, G., Marugán-Lobón, J., Chiappe, L. M., Sanz, J. L. & Buscalioni, Á. D. Soft-tissue and dermal arrangement in the wing of an Early Cretaceous bird: Implications for the evolution of avian flight. *Scientific Reports* **5**, 14864 (2015).

5 Xing, L. D. *et al.* Mummified precocial bird wings in mid-Cretaceous Burmese amber. *Nature Communications* **7**, 12089 (2016).

6 Zhou, Z., Chiappe, L. M. & Zhang, F. Anatomy of the Early Cretaceous bird *Eoenantiornis buhleri* (Aves: Enantiornithes) from China. *Canadian Journal of Earth Sciences* **42**, 1331-1338 (2005).
